# A Case Report of the Histologic Transformation of Primary Follicular Lymphoma of the Duodenum

**DOI:** 10.1097/MD.0000000000000165

**Published:** 2014-12-05

**Authors:** Shintaro Akiyama, Koji Izutsu, Yasunori Ota, Tsunao Imamura, Osamu Ogawa, Atsushi Wake, Kazuo Takeuchi

**Affiliations:** From the Department of Hematology (KI, AW); Department of Gastroenterology (SA, TI, OO, KT); Department of Pathology, Tokyo(YO); and Department of Hematology, Toranomon Hospital Kajigaya, Kawasaki, Japan (AW).

## Abstract

A 46-year-old woman underwent upper endoscopy for evaluation of anemia, which revealed whitish granules at the duodenal papilla, diagnosed as duodenal follicular lymphoma (DFL) by biopsy. Computed tomography and abdominal ultrasonography revealed that follicular lymphoma was confined to the duodenum. Seven years after the diagnosis, fluorine-18 fluorodeoxyglucose positron emission tomography scanning revealed multiple lesions including in bone marrow and lymph nodes. Bone marrow biopsy of the right iliac bone revealed diffuse large B-cell lymphoma, indicating systemic dissemination and histologic transformation of the DFL. The patient responded to chemotherapy and has been progression-free for 2.5 years. Although DFL is usually indolent even without any treatment, systemic dissemination with histologic transformation can occur. This case suggests that the life-time follow-up that is usually done for patients with nodal follicular lymphoma should be provided to patients with DFL.

## INTRODUCTION

Follicular lymphoma (FL) is a neoplasm of germinal center B cells and is the second most common subtype of non-Hodgkin lymphoma. Most patients with FL present with lymph node enlargement associated with bone marrow involvement. Recently, FL that affects the duodenum (DFL) has been recognized. DFL is reported to be localized around Vater papilla and consists of multiple small polyps. Primary DFL is an emerging clinical entity characterized by rare dissemination outside the gastrointestinal tract and by excellent prognosis. Because the disease frequently involves the jejunum and ileum, imaging of the entire small bowel by double-balloon enteroscopy or capsule endoscopy is encouraged.^1^ DFL has been recognized only recently and its features are not as yet fully understood.

It is possible for FL to undergo histologic transformation into diffuse large B-cell lymphoma (DLBCL). The reported incidence of histologic transformation varies, ranging from 16% to 60% depending on the length of follow-up and the rebiopsy/autopsy policy.^2,3^ Systemic progression associated with histologic transformation of DFL has rarely been reported in the literature. Here, we report a case of DFL that transformed into DLBCL.

## CASE PRESENTATION

**FIGURE 1:**
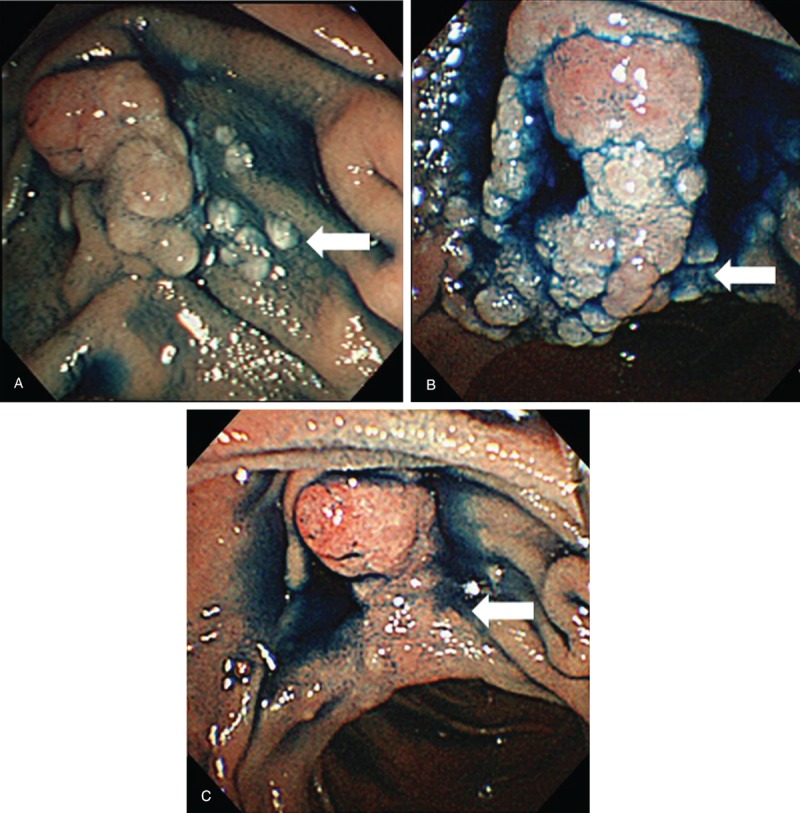
The series of endoscopic findings. At diagnosis of follicular lymphoma, the side-viewing endoscope revealed whitish granules around Vater papilla (white arrow) (A). At the time of transformation, whitish granules at Vater papilla increased in number and clustered together at Vater papilla (white arrow) (B). After 6 cycles of chemotherapy, the number of whitish granules decreased dramatically (white arrow) (C).

**FIGURE 2:**
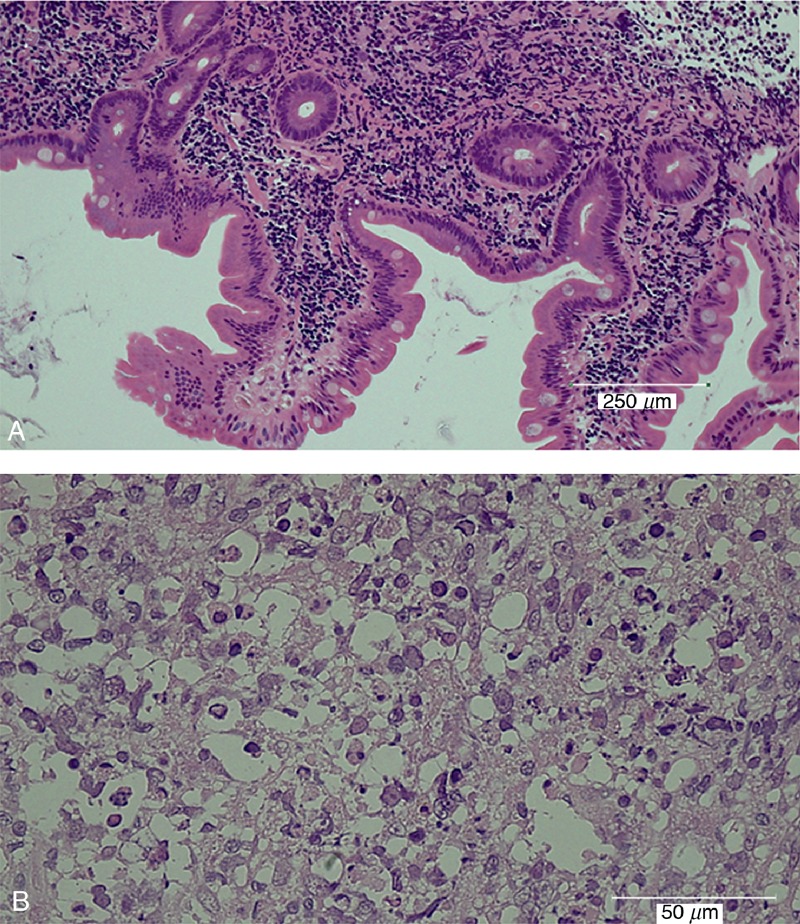
Endoscopic biopsy of granules around Vater papilla revealed the balloon-like villous hypertrophy caused by extrafollicular lymphoma cells (HE staining, Scale bar: 250 μm) (A). Biopsy of right iliac bone marrow showed sheets of large B cells (centroblasts), suggesting histologic transformation of follicular lymphoma into diffuse large B-cell lymphoma. Normal hematopoietic cells were replaced by lymphoma cells (HE staining, Scale bar: 50 μm) (B).

**FIGURE 3:**
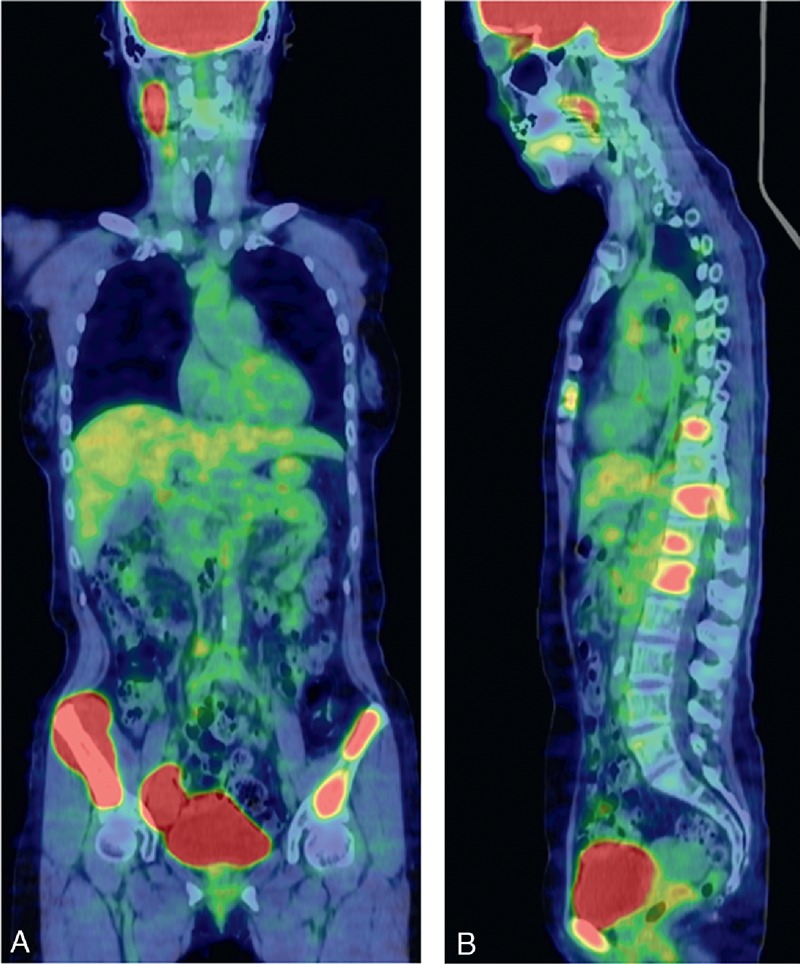
The result of ^18^F-FDG-PET scanning at transformation. ^18^F-FDG accumulated in the iliac bone, left pubic bone, thoracic vertebrae, right neck and flank, and right iliac lymph nodes. Red signals indicate accumulation of ^18^F-FDG, suggesting high tumor proliferation. The maximum standardized uptake value was 18.7 in the right iliac bone. (coronal view [A], sagittal view[B]). ^18^F-FDG-PET = fluorine-18 fluorodeoxyglucose positron emission tomography.

A 46-year-old female underwent upper endoscopy for evaluation of anemia. She was an asymptomatic hepatitis B virus (HBV) carrier but had no notable past medical history. Her family history was significant for a mother and an older brother with hepatitis B cirrhosis and hepatocellular carcinoma. On upper endoscopy, multiple whitish granules were seen around Vater papilla (Figure 1A). The biopsy specimen revealed nodular architecture of small lymphocytes and balloon-like villous hypertrophy caused by extrafollicular lymphoma cells (Figure 2A). Immunohistochemical staining revealed that lymphoma cells were positive for CD20, CD79a, CD10, BCL2, and BCL6 and were negative for CD3 and CD5. The MIB-1 positive cells accounted for approximately 10% of all cells. Fluorescence in situ hybridization analysis revealed the *IgH/BCL2* translocation, which was consistent with the diagnosis of follicular lymphoma. Lymphadenopathy was not detected by abdominal ultrasonography and computed tomography. On physical examination, there were no palpable superficial lymph nodes and no hepatosplenomegaly. Thus, she was diagnosed with DFL and was categorized as stage I according to the Lugano classification system. Hence, as her anemia was supposed to be not related to duodenal lesion, we chose to observe her without any local or systemic treatment. After 1.5 years of her DFL diagnosis, ultrasonography, and computed tomography revealed paraaortic lymph nodes 2 cm in diameter (Table 1). The patient was found to have a palpable lymph node 1 cm in diameter on the right neck 3 years after the diagnosis (Table 1). Annual upper endoscopy showed a gradually increasing number of whitish granules around Vater papilla (Figure 1B). Seven years after the diagnosis, laboratory tests revealed marked elevation of lactate dehydrogenase at 362 IU/L and soluble interleukin-2 receptor at 783 U/mL. Fluorine-18 fluorodeoxyglucose positron emission tomography (^18^F-FDG-PET) scanning was performed and revealed ^18^F-FDG accumulation in the iliac bone, left pubic bone, thoracic vertebrae, right neck and flank, and right iliac lymph nodes (Figure 3A and B). The standardized uptake value max was 18.7 for the right iliac lymph nodes. Despite the fact that the findings from the biopsy specimens from the duodenum and right neck lymph node were consistent with FL, the biopsy of right iliac bone marrow showed sheets of large centroblasts that were positive for CD20, CD10, BCL6, BCL2 and negative for CD5, MUM-1 (Figure 2B). The MIB-1 positive cells accounted for 80% of all cells. FISH analysis revealed the *IgH / BCL2* rearrangement. These findings were consistent with the diagnosis of DLBCL transformed from DFL. The Ann Arbor stage at transformation was IV.

**TABLE 1:**
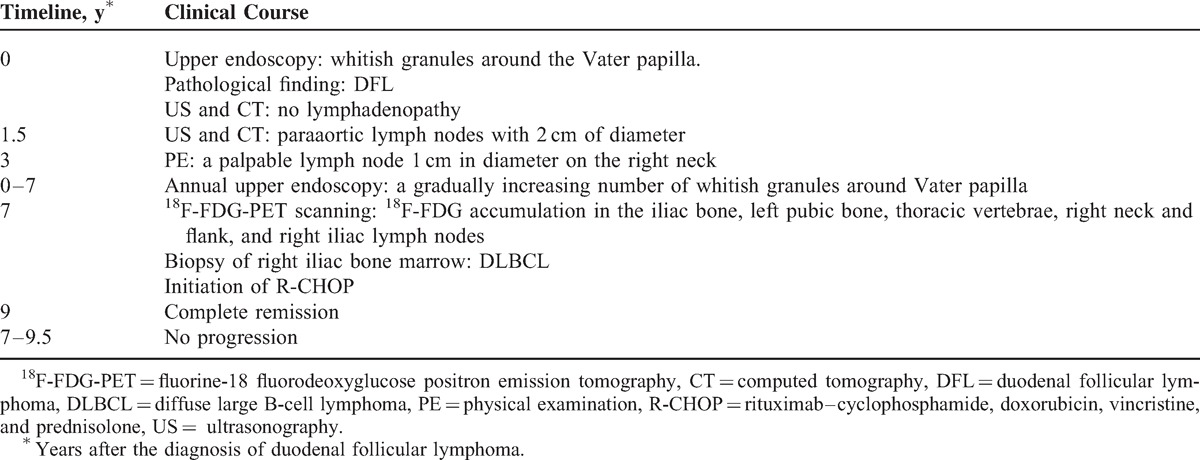
Timeline of Clinical Course

## THERAPEUTIC INTERVENTION

As chemotherapy with R-CHOP regimen, rituximab (375 mg/m^2^)–cyclophosphamide (750 mg/m^2^), doxorubicin (50 mg/m^2^), vincristine (1.4 mg/m^2^ (max 2.0 mg), and prednisolone (60 mg/m^2^) every 21 days, 6 cycles were administered. Entecavir 0.5 mg/day was initiated to prevent hepatitis B virus reactivation.

## FOLLOW-UP AND OUTCOMES

After the chemotherapy, upper endoscopy revealed the number of whitish granules dramatically were decreased (Figure 1C). The biopsy specimens of duodenal lesions revealed no infiltration of atypical lymphocytes. ^18^F-FDG-PET showed no ^18^F-FDG accumulation. Hence, complete remission of DLBCL transformed from DFL was achieved 9 years after the diagnosis (Table 1). The patient has been progression-free for 2.5 years after the transformation was diagnosed (Table 1).

## DISCUSSION

The most frequently involved gastrointestinal follicular lymphoma (GI-FL) site is the second portion of the duodenum (81%), followed by the jejunum (40%).^4^ Several lines of evidence suggest that GI-FL including DFL has distinct clinicopathological features from nodal FL. Unlike nodal FL, local progression, dissemination outside the small bowel, or transformation to aggressive lymphoma was virtually undetectable, even if left untreated, in a series of 63 patients with DFL.^1^ Moreover, DFL has been shown to share biological characteristics with mucosa-associated lymphoid tissue lymphoma as demonstrated by its association with follicular dendritic cell, immunoglobulin heavy chain variable region family usage, and gene expression profiling.^5–7^ However, clinical course and prognosis of patients with DFL still remain unclear.

In general, the decision to treat FL is made based on histology, presence, or absence of symptoms, disease burden, comorbidities, patient age, and patient preferences, with reference to the risks involved.^8^ Because FL usually progresses gradually, delayed treatment (“watch and wait” policy) is a reasonable option for patients having asymptomatic nodal FL with low tumor burden.^9^ The optimal treatment of GI-FL remains controversial. A “watch and wait” policy has been adopted in asymptomatic patients with GI-FL,^10^ for those with nodal FL with low tumor burden. Damaj et al^11^ reported that 7 GI-FL patients did not receive any treatment, and 4 of them progressed after a median follow-up of 37.5 months, but despite this, overall survival was excellent. However, surgery, radiotherapy, and chemotherapy are also applicable for other patients.^11^

Treatment of DFL is also controversial. According to a retrospective analysis of 63 cases of primary DFL, all of which were low grade (1 to 2), the initial therapies included watchful waiting (24 cases), radiotherapy (19 cases), rituximab monotherapy (5 cases), CHOP (2 cases), R-CHOP (2 cases), radiotherapy and rituximab (1 case), mitoxantrone, chlorambucil, and prednisolon (1 case), chlorambucil and prednisolone (1 case), and unknown regimen (1 case). Schmatz et al^1^ showed that a watch and wait approach for primary DFL appears to be the most sensible strategy. Mori et al^12^ described a series of 27 cases of primary DFL; 14 patients received therapy (local radiotherapy in 2 cases, and chemotherapy, including rituximab, in 12). Their estimated progression-free survival rate at 3 years was 70%. The other 13 cases did not receive treatment and their progression-free survival rate at 3 years was 74%. Therefore, due to the usually fairly indolent clinical course of primary DFL, a watch and wait policy would be appropriate initial management.^12^ In the current case, we chose to watch and wait because there was a risk of HBV reactivation after rituximab and/or chemotherapy. The patient was progression-free for 7 years after the diagnosis until histologic transformation occurred.

This patient was an asymptomatic HBV carrier. Recent meta-analysis revealed that HBV-infected people have higher risk for developing non-Hodgkin lymphoma with the odds ratios of HBV-infected population developing FL and DLBCL being 1.66 and 1.84, respectively.^13^ Biological mechanism responsible for lymphomagenesis in patients with HBV infection, however, is still unclear.

This case indicates that DFL can undergo histological transformation into DLBCL. There are few reports of the transformation of DFL into DLBCL. Sentani et al^14^ showed that, of 26 cases of DFL at various stages, 1 experienced transformation of DFL to DLBCL in a submandibular lymph node 4 months after the diagnosis of primary DFL. In addition, Mori et al^12^ reported that 1 of 27 cases of primary DFL developed histologic transformation. On the contrary, Schmatz et al^1^ reported that there was no transformation to high grade B-cell lymphoma among 63 cases of stage I DFL. It might be strange that histological transformation in this case was demonstrated in the bone marrow while the original lesion in the duodenum remained without transformation. Recently, using whole-exome sequencing, Pasqualucci L et al^15^ beautifully demonstrated that transformation of FL does not evolve as a linear process (emergence of an aggressive subclone from the initial dominant FL clone) but derives from the divergent evolution of an ancestral common precursor cell that acquired distinct mutations to become a FL or a transformed FL. Although bone marrow involvement had not been demonstrated at diagnosis in this patient, it might be possible that common precursor cell exists in the bone marrow.

In conclusion, although it is rare, transformation from DFL to DLBCL can occur. Thus, life-long follow-up with timely imaging, as is done for patients with nodal FL, is required for patients with DFL.
